# Supplementary material for: W-state Analyzer and Multi-party Measurement-device-independent Quantum Key Distribution
Source: Sci Rep. 2015 Dec 8;5:17449. doi: 10.1038/srep17449 (PMC4672340; doi:10.1038/srep17449)
Supplement: Supplementary Information [file srep17449-s1.doc]

**Supplementary materials: W-state Analyzer and Multi-party Measurement-device-independent Quantum Key Distribution**

**Changhua Zhu^1 2*^, Feihu Xu^3^, & Changxing Pei^1^**

^1^State Key Laboratory of Integrated Services Networks, Xidian University, Xi’an, Shaanxi 710071, China

^2^Department of Electrical & Computer Engineering, University of Toronto, Toronto, Ontario M5S 3G4, Canada

^3^Research Laboratory of Electronics, Massachusetts Institute of Technology, Cambridge, Massachusetts 02139, USA

*Corresponding author: chhzhu@xidian.edu.cn

**I. 16 $W_{4}$ states**

The 16 $W_{4}$ states are given as follows:

$|W_{\text{4,} 0}\rangle=1/2\left(|0001\rangle+|0010\rangle+|0100\rangle+|1000\rangle\right)$ (S1)

$|W_{\text{4,} 1}\rangle=1/2\left(|0001\rangle-|0010\rangle-|0100\rangle+|1000\rangle\right)$ (S2)

$|W_{\text{4,} 2}\rangle=1/2\left(|0001\rangle-|0010\rangle+|0100\rangle-|1000\rangle\right)$ (S3)

$|W_{\text{4,} 3}\rangle=1/2\left(|0001\rangle+|0010\rangle-|0100\rangle-|1000\rangle\right)$ (S4)

$|W_{\text{4,} 4}\rangle=1/2\left(|0000\rangle+|1100\rangle+|1010\rangle+|1001\rangle\right)$ (S5)

$|W_{\text{4,} 5}\rangle=1/2\left(|0000\rangle-|1100\rangle-|1010\rangle+|1001\rangle\right)$ (S6)

$|W_{\text{4,} 6}\rangle=1/2\left(|0000\rangle-|1100\rangle+|1010\rangle-|1001\rangle\right)$ (S7)

$|W_{\text{4,} 7}\rangle=1/2\left(|0000\rangle+|1100\rangle-|1010\rangle-|1001\rangle\right)$ (S8)

$|W_{\text{4,} 8}\rangle=1/2\left(|0011\rangle+|0101\rangle+|0110\rangle+|1111\rangle\right)$ (S9)

$|W_{\text{4,} 9}\rangle=1/2\left(|0011\rangle-|0101\rangle-|0110\rangle+|1111\rangle\right)$ (S10)

$|W_{\text{4,} \text{a}}\rangle=1/2\left(|0011\rangle-|0101\rangle+|0110\rangle-|1111\rangle\right)$ (S11)

$|W_{\text{4,} \text{b}}\rangle=1/2\left(|0011\rangle+|0101\rangle-|0110\rangle-|1111\rangle\right)$ (S12)

$|W_{\text{4,} \text{c}}\rangle=1/2\left(|0111\rangle+|1011\rangle+|1101\rangle+|1110\rangle\right)$ (S13)

$|W_{\text{4,} \text{d}}\rangle=1/2\left(|0111\rangle-|1011\rangle-|1101\rangle+|1110\rangle\right)$ (S14)

$|W_{\text{4,} \text{e}}\rangle=1/2\left(|0111\rangle-|1011\rangle+|1101\rangle-|1110\rangle\right)$ (S15)

$|W_{\text{4,} \text{f}}\rangle=1/2\left(|0111\rangle+|1011\rangle-|1101\rangle-|1110\rangle\right)$ (S16)

The state $|W_{\text{4, 0}}\rangle$ given by equation (S1) is a standard $W_{4}$ state and belongs to one family states defined in Ref. 1. The states $|W_{\text{4, 1}}\rangle$, $|W_{\text{4, 2}}\rangle$ and $|W_{\text{4, 3}}\rangle$ can be constructed by performing unitary operations $IZZI$, $ZIZI$ and $ZZII$ on state $|W_{\text{4, 0}}\rangle$, respectively, where $I$ is the unit operator and $Z$ is the Pauli $Z$ operator. The states $|W_{\text{4, c}}\rangle$, $|W_{\text{4, d}}\rangle$, $|W_{\text{4, e}}\rangle$and $|W_{\text{4, f}}\rangle$ can be constructed by performing $XXXX$ operations on the states $|W_{\text{4, 0}}\rangle$, $|W_{\text{4, 1}}\rangle$, $|W_{\text{4, 2}}\rangle$ and $|W_{\text{4, 3}}\rangle$, respectively, where $X$ is the Pauli $X$ operator. The state $|W_{\text{4, 4}}\rangle$ can be obtained by performing unitary operation U

$$U=\left[\begin{matrix} 0 & 1 & 0 & 0 & 0 & 0 & 0 & 0 & 0 & 0 & 0 & 0 & 0 & 0 & 0 & 0 \\ 1 & 0 & 0 & 0 & 0 & 0 & 0 & 0 & 0 & 0 & 0 & 0 & 0 & 0 & 0 & 0 \\ 0 & 0 & 0 & 1 & 0 & 0 & 0 & 0 & 0 & 0 & 0 & 0 & 0 & 0 & 0 & 0 \\ 0 & 0 & 0 & 0 & 0 & 1 & 0 & 0 & 0 & 0 & 0 & 0 & 0 & 0 & 0 & 0 \\ 0 & 0 & 0 & 0 & 0 & 0 & 1 & 0 & 0 & 0 & 0 & 0 & 0 & 0 & 0 & 0 \\ 0 & 0 & 0 & 0 & 0 & 0 & 0 & 1 & 0 & 0 & 0 & 0 & 0 & 0 & 0 & 0 \\ 0 & 0 & 0 & 0 & 0 & 0 & 0 & 0 & 0 & 1 & 0 & 0 & 0 & 0 & 0 & 0 \\ 0 & 0 & 0 & 0 & 0 & 0 & 0 & 0 & 0 & 0 & 1 & 0 & 0 & 0 & 0 & 0 \\ 0 & 0 & 0 & 0 & 0 & 0 & 0 & 0 & 0 & 0 & 0 & 1 & 0 & 0 & 0 & 0 \\ 0 & 0 & 1 & 0 & 0 & 0 & 0 & 0 & 0 & 0 & 0 & 0 & 0 & 0 & 0 & 0 \\ 0 & 0 & 0 & 0 & 1 & 0 & 0 & 0 & 0 & 0 & 0 & 0 & 0 & 0 & 0 & 0 \\ 0 & 0 & 0 & 0 & 0 & 0 & 0 & 0 & 0 & 0 & 0 & 0 & 1 & 0 & 0 & 0 \\ 0 & 0 & 0 & 0 & 0 & 0 & 0 & 0 & 1 & 0 & 0 & 0 & 0 & 0 & 0 & 0 \\ 0 & 0 & 0 & 0 & 0 & 0 & 0 & 0 & 0 & 0 & 0 & 0 & 0 & 1 & 0 & 0 \\ 0 & 0 & 0 & 0 & 0 & 0 & 0 & 0 & 0 & 0 & 0 & 0 & 0 & 0 & 1 & 0 \\ 0 & 0 & 0 & 0 & 0 & 0 & 0 & 0 & 0 & 0 & 0 & 0 & 0 & 0 & 0 & 1 \end{matrix}\right]$$

on $|W_{\text{4, 0}}\rangle$. The states $|W_{\text{4, 5}}\rangle$, $|W_{\text{4, 6}}\rangle$and $|W_{\text{4, 7}}\rangle$ can be obtained by performing $ZIIZ$, $ZIZI$ and $ZZII$ operations on state $|W_{\text{4, 4}}\rangle$, respectively. The state $|W_{\text{4, 8}}\rangle$ can be obtained by performing $XXXX$ operation on state $|W_{\text{4, 4}}\rangle$. The state $|W_{\text{4, 9}}\rangle$ can be obtained by performing $IIZZ$operation on state $|W_{\text{4, 8}}\rangle$. The states $|W_{\text{4, a}}\rangle$ and $|W_{\text{4, b}}\rangle$ can be obtained by performing $-XXXX$on states $|W_{\text{4, 6}}\rangle$ and $|W_{\text{4, 5}}\rangle$, respectively. All these $W_{4}$ states form a group of orthogonal bases in the 16-dimensional Hilbert space. Any 4-qubit state can be expressed as a linear combination of these 16 $W_{4}$ states.

**II. Details of W-state preparation based on entanglement swapping**

Let the 4 Bell states prepared by Alice, Bob, Charlie and David are , $1/\sqrt{2}\left(|00\rangle_{BB'}+|11\rangle_{BB'}\right)$, and , respectively. The state of the system is

$\begin{matrix} |\psi_{S}\rangle=1/4\left(|00\rangle_{AA'}+|11\rangle_{AA'}\right)\left(|00\rangle_{BB'}+|11\rangle_{BB'}\right)\left(|00\rangle_{CC'}+|11\rangle_{CC'}\right)\left(|00\rangle_{DD'}+|11\rangle_{DD'}\right) \\ \begin{matrix} & \end{matrix}=1/4\left\{|0000\rangle_{ABCD}|0000\rangle_{A'B'C'D'}+|0001\rangle_{ABCD}|0001\rangle_{A'B'C'D'}+|0010\rangle_{ABCD}|0010\rangle_{A'B'C'D'}+\right. \\ \begin{matrix} & & \end{matrix}+|0011\rangle_{ABCD}|0011\rangle_{A'B'C'D'}+|0100\rangle_{ABCD}|0100\rangle_{A'B'C'D'}+|0101\rangle_{ABCD}|0101\rangle_{A'B'C'D'}+ \\ \begin{matrix} & & \end{matrix}+|0110\rangle_{ABCD}|0110\rangle_{A'B'C'D'}+|0111\rangle_{ABCD}|0111\rangle_{A'B'C'D'}+|1000\rangle_{ABCD}|1000\rangle_{A'B'C'D'}+ \\ \begin{matrix} & & \end{matrix}+|1001\rangle_{ABCD}|1001\rangle_{A'B'C'D'}+|1010\rangle_{ABCD}|1010\rangle_{A'B'C'D'}+|1011\rangle_{ABCD}|1011\rangle_{A'B'C'D'}+ \\ \begin{matrix} & & \end{matrix}+|1100\rangle_{ABCD}|1100\rangle_{A'B'C'D'}+|1101\rangle_{ABCD}|1101\rangle_{A'B'C'D'}+|1110\rangle_{ABCD}|1110\rangle_{A'B'C'D'}+ \\ \begin{matrix} & & \end{matrix}\left.+|1111\rangle_{ABCD}|1111\rangle_{A'B'C'D'}\right\} \end{matrix}$ (S17)

The 16 basis states can be represented by 16 $W_{4}$ states as follows

$|0001\rangle_{A'B'C'D'}=1/2\left(|W_{4,0}\rangle+|W_{4,1}\rangle+|W_{4,2}\rangle+|W_{4,3}\rangle\right)_{A'B'C'D'}$ (S18)

$|0010\rangle_{A'B'C'D'}=1/2\left(|W_{4,0}\rangle-|W_{4,1}\rangle-|W_{4,2}\rangle+|W_{4,3}\rangle\right)_{A'B'C'D'}$ (S19)

$|0100\rangle_{A'B'C'D'}=1/2\left(|W_{4,0}\rangle-|W_{4,1}\rangle+|W_{4,2}\rangle-|W_{4,3}\rangle\right)_{A'B'C'D'}$ (S20)

$|1000\rangle_{A'B'C'D'}=1/2\left(|W_{4,0}\rangle+|W_{4,1}\rangle-|W_{4,2}\rangle-|W_{4,3}\rangle\right)_{A'B'C'D'}$ (S21)

$|0000\rangle_{A'B'C'D'}=1/2\left(|W_{4,4}\rangle+|W_{4,5}\rangle+|W_{4,6}\rangle+|W_{4,7}\rangle\right)_{A'B'C'D'}$ (S22)

$|1100\rangle_{A'B'C'D'}=1/2\left(|W_{4,4}\rangle-|W_{4,5}\rangle-|W_{4,6}\rangle+|W_{4,7}\rangle\right)_{A'B'C'D'}$ (S23)

$|1010\rangle_{A'B'C'D'}=1/2\left(|W_{4,4}\rangle-|W_{4,5}\rangle+|W_{4,6}\rangle-|W_{4,7}\rangle\right)_{A'B'C'D'}$ (S24)

$|1001\rangle_{A'B'C'D'}=1/2\left(|W_{4,4}\rangle+|W_{4,5}\rangle-|W_{4,6}\rangle-|W_{4,7}\rangle\right)_{A'B'C'D'}$ (S25)

$|0011\rangle_{A'B'C'D'}=1/2\left(|W_{4,8}\rangle+|W_{4,9}\rangle+|W_{4,a}\rangle+|W_{4,b}\rangle\right)_{A'B'C'D'}$ (S26)

$|0101\rangle_{A'B'C'D'}=1/2\left(|W_{4,8}\rangle-|W_{4,9}\rangle-|W_{4,a}\rangle+|W_{4,b}\rangle\right)_{A'B'C'D'}$ (S27)

$|0110\rangle_{A'B'C'D'}=1/2\left(|W_{4,8}\rangle-|W_{4,9}\rangle+|W_{4,a}\rangle-|W_{4,b}\rangle\right)_{A'B'C'D'}$ (S28)

$|1111\rangle_{A'B'C'D'}=1/2\left(|W_{4,8}\rangle+|W_{4,9}\rangle-|W_{4,a}\rangle-|W_{4,b}\rangle\right)_{A'B'C'D'}$ (S29)

$|0111\rangle_{A'B'C'D'}=1/2\left(|W_{4,c}\rangle+|W_{4,d}\rangle+|W_{4,e}\rangle+|W_{4,f}\rangle\right)_{A'B'C'D'}$ (S30)

$|1011\rangle_{A'B'C'D'}=1/2\left(|W_{4,c}\rangle-|W_{4,d}\rangle-|W_{4,e}\rangle+|W_{4,f}\rangle\right)_{A'B'C'D'}$ (S31)

$|1101\rangle_{A'B'C'D'}=1/2\left(|W_{4,c}\rangle-|W_{4,d}\rangle+|W_{4,e}\rangle-|W_{4,f}\rangle\right)_{A'B'C'D'}$ (S32)

$|1110\rangle_{A'B'C'D'}=1/2\left(|W_{4,c}\rangle+|W_{4,d}\rangle-|W_{4,e}\rangle-|W_{4,f}\rangle\right)_{A'B'C'D'}$ (S33)

Then,

$\begin{matrix} |\psi_{S}\rangle=1/4\left\{|W_{4,0}\rangle_{ABCD}|W_{4,0}\rangle_{A'B'C'D'}+|W_{4,1}\rangle_{ABCD}|W_{4,1}\rangle_{A'B'C'D'}+|W_{4,2}\rangle_{ABCD}|W_{4,2}\rangle_{A'B'C'D'}+\right. \\ \begin{matrix} & & \end{matrix}|W_{4,3}\rangle_{ABCD}|W_{4,3}\rangle_{A'B'C'D'}+|W_{4,4}\rangle_{ABCD}|W_{4,4}\rangle_{A'B'C'D'}+|W_{4,5}\rangle_{ABCD}|W_{4,5}\rangle_{A'B'C'D'}+ \\ \begin{matrix} & & \end{matrix}|W_{4,6}\rangle_{ABCD}|W_{4,6}\rangle_{A'B'C'D'}+|W_{4,7}\rangle_{ABCD}|W_{4,7}\rangle_{A'B'C'D'}+|W_{4,8}\rangle_{ABCD}|W_{4,8}\rangle_{A'B'C'D'}+ \\ \begin{matrix} & & \end{matrix}|W_{4,9}\rangle_{ABCD}|W_{4,9}\rangle_{A'B'C'D'}+|W_{4,a}\rangle_{ABCD}|W_{4,a}\rangle_{A'B'C'D'}+|W_{4,b}\rangle_{ABCD}|W_{4,b}\rangle_{A'B'C'D'}+ \\ \begin{matrix} & & \end{matrix}|W_{4,c}\rangle_{ABCD}|W_{4,c}\rangle_{A'B'C'D'}+|W_{4,d}\rangle_{ABCD}|W_{4,d}\rangle_{A'B'C'D'}+|W_{4,e}\rangle_{ABCD}|W_{4,e}\rangle_{A'B'C'D'}+ \\ \begin{matrix} & & \end{matrix}\left.|W_{4,f}\rangle_{ABCD}|W_{4,f}\rangle_{A'B'C'D'}\right\} \end{matrix}$ (S34)

Therefore, from equation (S34) we obtain that when the state of particles $A',B',C'$ and $D'$ are projected into one of the $W_{4}$ states, the state of particles $A,B,C$ and $D$ will be in the corresponding $W_{4}$ state.

**III. Details of estimation of** $Q_{1}$ **and** $e_{1}$

We compute the gains at different cases, respectively.

(1) Case 1: outputs deriving from four background counts

In this case, there is no successful photon transmission (with probability $\left(1-\eta_{a}\right)\left(1-\eta_{b}\right)\left(1-\eta_{c}\right)\left(1-\eta_{d}\right)$, where $\eta_{i}$is the transmittance of participant $i$, $i=a,b,c,d$). Emma will give an output when there exist dark counts in four specific modes according to Table 1 (with probability $Y_{0}^{4}$) and there are no dark counts in other 12 modes (with probability $\left(1-Y_{0}\right)^{12}$ ) . There are 12 (4) detection modes for states $|W_{4,0}\rangle$ and $|W_{4,c}\rangle$ ($|W_{4,1}\rangle$ and $|W_{4,d}\rangle$). Based on the post-selection scheme shown in Table 2, input states$0000, 0001, 0010$ and $0011$(corresponding to the states $|W_{4,0}\rangle$ and $|W_{4,1}\rangle$), $1100, 1101, 1110$ and $1111$ (corresponding to $|W_{4,c}\rangle$ and $|W_{4,d}\rangle$) can be used to generate key. Each input state ($0000, 0001, ..., 1110$ or $1111$) is prepared with equal probability, i.e. each one with probability $1/16$. So the gain at this case is

$\begin{matrix} 1/16\left\{4\left(1-\eta_{a}\right)\left(1-\eta_{b}\right)\left(1-\eta_{c}\right)\left(1-\eta_{d}\right)\left[12Y_{0}^{4}\left(1-Y_{0}\right)^{12}\text{+4}Y_{0}^{4}\left(1-Y_{0}\right)^{12}\right]\right.\text{+} \\ \begin{matrix} & \end{matrix}\left.4\left(1-\eta_{a}\right)\left(1-\eta_{b}\right)\left(1-\eta_{c}\right)\left(1-\eta_{d}\right)\left[12Y_{0}^{4}\left(1-Y_{0}\right)^{12}\text{+4}Y_{0}^{4}\left(1-Y_{0}\right)^{12}\right]\right\} \\ =8\left(1-\eta_{a}\right)\left(1-\eta_{b}\right)\left(1-\eta_{c}\right)\left(1-\eta_{d}\right)Y_{0}^{4}\left(1-Y_{0}\right)^{12} \end{matrix}$ (S35)

While, qubit error will occur when input states are $\text{0000,} \text{0011,} 1100$ and $1111$ with probability

$4\left(1-\eta_{a}\right)\left(1-\eta_{b}\right)\left(1-\eta_{c}\right)\left(1-\eta_{d}\right)Y_{0}^{4}\left(1-Y_{0}\right)^{12}$. (S36)

(2) Case 2: outputs deriving from one photon count and three background counts

In this case, one count derives from a successful photon transmission and the other three counts derive from background. For an input photon, it can arrive at the SPD in different spatial and temporal modes, e.g. the state $a_{a,t_{0}}^{\dagger}|0\rangle$ of Alice’s photon can be mapped into the state

$\begin{matrix} a_{a,t_{0}}^{\dagger}|0\rangle\to1/\left(4\sqrt{2}\right)\left(-2a_{s,t_{0}}^{\dagger}-2e^{i\delta}a_{s,t_{1}}^{\dagger}+2ie^{i\delta}a_{u,t_{1}}^{\dagger}+2ie^{i2\delta}a_{u,t_{2}}^{\dagger}-2ia_{v,t_{0}}^{\dagger}\right. \\ \begin{matrix} & & \end{matrix}\left.+2ie^{i\delta}a_{v,t_{1}}^{\dagger}-2e^{i\delta}a_{w,t_{1}}^{\dagger}+2e^{i2\delta}a_{w,t_{2}}^{\dagger}\right)|0\rangle\end{matrix}$, (S37)

and the state $a_{a,t_{1}}^{\dagger}|0\rangle$ of Alice’s photon can be mapped into the state

$\begin{matrix} a_{a,t_{1}}^{\dagger}|0\rangle\to1/\left(4\sqrt{2}\right)\left(-2a_{s,t_{1}}^{\dagger}-2e^{i\delta}a_{s,t_{2}}^{\dagger}+2ie^{i\delta}a_{u,t_{2}}^{\dagger}+2ie^{i2\delta}a_{u,t_{3}}^{\dagger}-2ia_{v,t_{1}}^{\dagger}\right. \\ \begin{matrix} & & \end{matrix}\left.+2ie^{i\delta}a_{v,t_{2}}^{\dagger}-2e^{i\delta}a_{w,t_{2}}^{\dagger}+2e^{i2\delta}a_{w,t_{3}}^{\dagger}\right)|0\rangle\end{matrix}$ . (S38)

When Alice’s photon, e.g. in state $a_{a,t_{0}}^{\dagger}|0\rangle$, is detected and the input state is $|0000\rangle$, the output probability of Emma’s device according to Table 1 is

$\begin{matrix} \eta_{a}\left(1-\eta_{b}\right)\left(1-\eta_{c}\right)\left(1-\eta_{d}\right)\left(1/4/\sqrt{2}\right)^{2}\left[2^{2}\times10+2^{2}\times3\right.+2^{2}\times10+2^{2}\times3+ \\ \begin{matrix} & \end{matrix}\left.2^{2}\times10+2^{2}\times3+2^{2}\times10+2^{2}\times3\right]Y_{0}^{3}\left(1-Y_{0}\right)^{12} \\ \begin{matrix} & \end{matrix}=208/32\eta_{a}\left(1-\eta_{b}\right)\left(1-\eta_{c}\right)\left(1-\eta_{d}\right)Y_{0}^{3}\left(1-Y_{0}\right)^{12} \end{matrix}$ (S39)

In equation (S39), 10 denotes the number of operators $a_{s,t_{0}}^{\dagger},a_{u,t_{1}}^{\dagger},a_{v,t_{0}}^{\dagger}a_{w,t_{1}}^{\dagger}$in the detection modes of the W states $|W_{4,0}\rangle$ and $|W_{4,1}\rangle$; 3 denotes the number of operators $a_{s,t_{1}}^{\dagger},a_{u,t_{2}}^{\dagger},a_{v,t_{1}}^{\dagger},a_{w,t_{2}}^{\dagger}$ in the detection modes of the W states $|W_{4,0}\rangle$ and $|W_{4,1}\rangle$. Similarly, we can obtain the output probability of other participants’ photons’ counts under different inputs state. So, the gain in this case is

$\begin{matrix} 1/16\left\{8\times208/32\left[\eta_{a}\left(1-\eta_{b}\right)\left(1-\eta_{c}\right)\left(1-\eta_{d}\right)+\eta_{b}\left(1-\eta_{a}\right)\left(1-\eta_{c}\right)\left(1-\eta_{d}\right)\right]\right.+ \\ \begin{matrix} & \end{matrix}\left(4\times208/32+4\times96/32\right)\left.\left[\eta_{c}\left(1-\eta_{a}\right)\left(1-\eta_{b}\right)\left(1-\eta_{d}\right)+\eta_{d}\left(1-\eta_{a}\right)\left(1-\eta_{b}\right)\left(1-\eta_{c}\right)\right]\right\}Y_{0}^{3}\left(1-Y_{0}\right)^{12} \\ \begin{matrix} & = \end{matrix}1/16\left\{52\left[\eta_{a}\left(1-\eta_{b}\right)\left(1-\eta_{c}\right)\left(1-\eta_{d}\right)+\eta_{b}\left(1-\eta_{a}\right)\left(1-\eta_{c}\right)\left(1-\eta_{d}\right)\right]\right.+ \\ \begin{matrix} & \end{matrix}38\left.\left[\eta_{c}\left(1-\eta_{a}\right)\left(1-\eta_{b}\right)\left(1-\eta_{d}\right)+\eta_{d}\left(1-\eta_{a}\right)\left(1-\eta_{b}\right)\left(1-\eta_{c}\right)\right]\right\}Y_{0}^{3}\left(1-Y_{0}\right)^{12} \end{matrix}$ (S40)

In this case the error will occur with probability

$\begin{matrix} 1/16\left\{26\left[\eta_{a}\left(1-\eta_{b}\right)\left(1-\eta_{c}\right)\left(1-\eta_{d}\right)+\eta_{b}\left(1-\eta_{a}\right)\left(1-\eta_{c}\right)\left(1-\eta_{d}\right)\right]\right.+ \\ \begin{matrix} & \end{matrix}19\left.\left[\eta_{c}\left(1-\eta_{a}\right)\left(1-\eta_{b}\right)\left(1-\eta_{d}\right)+\eta_{d}\left(1-\eta_{a}\right)\left(1-\eta_{b}\right)\left(1-\eta_{c}\right)\right]\right\}Y_{0}^{3}\left(1-Y_{0}\right)^{12} \end{matrix}$ (S41)

(3) Case 3: outputs deriving from two photon counts and two background counts

In this case, two counts derive from successful photon transmission and two counts derive from background. Any state of two input photons can evolve into a state superposed by different spatial and time modes, e.g. state $a_{a,t_{0}}^{\dagger}a_{b,t_{0}}^{\dagger}|0\rangle$ of Alice’s and Bob’s photons evolves into the state

$\begin{matrix} a_{a,t_{0}}^{\dagger}a_{b,t_{0}}^{\dagger}|0\rangle\to1/\left(2^{2}\cdot2^{2}\cdot2\right)\left[-i4e^{i4\delta}\left(a_{u,t_{2}}^{\dagger}\right)^{2}-8e^{i4\delta}a_{u,t_{2}}^{\dagger}a_{w,t_{2}}^{\dagger}+i4e^{i4\delta}\left(a_{w,t_{2}}^{\dagger}\right)^{2}+\right. \\ \begin{matrix} & & \end{matrix}8e^{i3\delta}a_{s,t_{1}}^{\dagger}a_{u,t_{2}}^{\dagger}-i8e^{i3\delta}a_{s,t_{1}}^{\dagger}a_{w,t_{2}}^{\dagger}-i8e^{i3\delta}a_{u,t_{2}}^{\dagger}a_{v,t_{1}}^{\dagger}-8e^{i3\delta}a_{v,t_{1}}^{\dagger}a_{w,t_{2}}^{\dagger}+i4e^{i2\delta}\left(a_{s,t_{1}}^{\dagger}\right)^{2}+ \\ \begin{matrix} & & \end{matrix}8e^{i2\delta}a_{s,t_{1}}^{\dagger}a_{v,t_{1}}^{\dagger}+i4e^{i2\delta}\left(a_{u,t_{1}}^{\dagger}\right)^{2}-8e^{i2\delta}a_{u,t_{1}}^{\dagger}a_{w,t_{1}}^{\dagger}-i4e^{i2\delta}\left(a_{v,t_{1}}^{\dagger}\right)^{2}-i4e^{i2\delta}\left(a_{w,t_{1}}^{\dagger}\right)^{2}- \\ \begin{matrix} & & \end{matrix}8e^{i\delta}a_{s,t_{0}}^{\dagger}a_{u,t_{1}}^{\dagger}-i8e^{i\delta}a_{s,t_{0}}^{\dagger}a_{w,t_{1}}^{\dagger}-i8e^{i\delta}a_{u,t_{1}}^{\dagger}a_{v,t_{0}}^{\dagger}+8e^{i\delta}a_{v,t_{0}}^{\dagger}a_{w,t_{1}}^{\dagger}-i4\left(a_{s,t_{0}}^{\dagger}\right)^{2}+ \\ \begin{matrix} & & \end{matrix}\left.8a_{s,t_{0}}^{\dagger}a_{v,t_{0}}^{\dagger}+i4\left(a_{v,t_{0}}^{\dagger}\right)^{2}\right]|0\rangle\end{matrix}$ (S42)

From Table 1, we can obtain that there are 6, 6, 4, 4, 6 and 6 detection modes for states $a_{s,t_{0}}^{\dagger}a_{u,t_{1}}^{\dagger},a_{s,t_{0}}^{\dagger}a_{v,t_{0}}^{\dagger},a_{s,t_{0}}^{\dagger}a_{w,t_{1}}^{\dagger},a_{u,t_{1}}^{\dagger}a_{v,t_{0}}^{\dagger},a_{u,t_{1}}^{\dagger}a_{w,t_{1}}^{\dagger}$and $a_{v,t_{0}}^{\dagger}a_{w,t_{1}}^{\dagger}$, respectively, corresponding to the states $|W_{4,0}\rangle$ and $|W_{4,1}\rangle$. Based on equation (S42) the output probability is

$\begin{matrix} \left(1/32\right)^{2}\eta_{a}\eta_{b}\left(1-\eta_{c}\right)\left(1-\eta_{d}\right)\times64\left(6+6+4+4+6+6\right)Y_{0}^{2}\left(1-Y_{0}\right)^{12} \\ \begin{matrix} & \end{matrix}=2\eta_{a}\eta_{b}\left(1-\eta_{c}\right)\left(1-\eta_{d}\right)Y_{0}^{2}\left(1-Y_{0}\right)^{12} \end{matrix}$ (S43)

Similarly, we can obtain the output probability of other participants’ photons counts under different inputs state. So, the gain in this case is

$\begin{matrix} 1/16\left\{16\eta_{a}\eta_{b}\left(1-\eta_{c}\right)\left(1-\eta_{d}\right)\right.+10\left[\eta_{a}\eta_{c}\left(1-\eta_{b}\right)\left(1-\eta_{d}\right)+\eta_{b}\eta_{d}\left(1-\eta_{a}\right)\left(1-\eta_{c}\right)\right]+ \\ \begin{matrix} & \end{matrix}9\left[\eta_{a}\eta_{d}\left(1-\eta_{b}\right)\left(1-\eta_{c}\right)+\eta_{b}\eta_{c}\left(1-\eta_{a}\right)\left(1-\eta_{d}\right)\right]+\left.8\eta_{c}\eta_{d}\left(1-\eta_{a}\right)\left(1-\eta_{b}\right)\right\}Y_{0}^{2}\left(1-Y_{0}\right)^{12} \end{matrix}$ (S44)

In this case the error will occur with probability

$\begin{matrix} 1/32\left\{16\eta_{a}\eta_{b}\left(1-\eta_{c}\right)\left(1-\eta_{d}\right)\right.+10\left[\eta_{a}\eta_{c}\left(1-\eta_{b}\right)\left(1-\eta_{d}\right)+\eta_{b}\eta_{d}\left(1-\eta_{a}\right)\left(1-\eta_{c}\right)\right]+ \\ \begin{matrix} & \end{matrix}9\left[\eta_{a}\eta_{d}\left(1-\eta_{b}\right)\left(1-\eta_{c}\right)+\eta_{b}\eta_{c}\left(1-\eta_{a}\right)\left(1-\eta_{d}\right)\right]+\left.8\eta_{c}\eta_{d}\left(1-\eta_{a}\right)\left(1-\eta_{b}\right)\right\}Y_{0}^{2}\left(1-Y_{0}\right)^{12} \end{matrix}$ (S45)

(4) Case 4: outputs deriving from three photon counts and one background count

In this case, three counts derive from successful photon transmission and one count derives from background. Any state of three input photons can evolve into a state superposed by different spatial and time modes, e.g. the state $a_{a,t_{0}}^{\dagger}a_{b,t_{0}}^{\dagger}a_{c,t_{0}}^{\dagger}|0\rangle$ of Alice’s, Bob’s and Charlie’s photons evolves into the state (partial terms are omitted for simplification)

$\begin{matrix} a_{a,t_{0}}^{\dagger}a_{b,t_{0}}^{\dagger}a_{c,t_{0}}^{\dagger}|0\rangle\to1/\left(2^{3}\cdot2^{3}\cdot\sqrt{2^{3}}\right)\left[8e^{i6\delta}\left(a_{u,t_{2}}^{\dagger}\right)^{3}-i8e^{i6\delta}\left(a_{u,t_{2}}^{\dagger}\right)^{2}a_{w,t_{2}}^{\dagger}+8e^{i6\delta}a_{u,t_{2}}^{\dagger}\left(a_{w,t_{2}}^{\dagger}\right)^{2}-\right. \\ \begin{matrix} & & \end{matrix}i8e^{i6\delta}\left(a_{w,t_{2}}^{\dagger}\right)^{3}+i8e^{i5\delta}a_{s,t_{1}}^{\dagger}\left(a_{u,t_{2}}^{\dagger}\right)^{2}-16e^{i5\delta}a_{s,t_{1}}^{\dagger}a_{u,t_{2}}^{\dagger}a_{w,t_{2}}^{\dagger}+i24e^{i5\delta}a_{s,t_{1}}^{\dagger}\left(a_{w,t_{2}}^{\dagger}\right)^{2}+ \\ \begin{matrix} & & \end{matrix} ... +i8e^{i\delta}\left(a_{v,t_{0}}^{\dagger}\right)^{2}a_{w,t_{1}}^{\dagger}+i8\left(a_{s,t_{0}}^{\dagger}\right)^{3}-8\left(a_{s,t_{0}}^{\dagger}\right)^{2}a_{v,t_{0}}^{\dagger}+i8a_{s,t_{0}}^{\dagger}\left(a_{v,t_{0}}^{\dagger}\right)^{2}-\left.8\left(a_{v,t_{0}}^{\dagger}\right)^{3}\right]|0\rangle\end{matrix}$ (S46)

In equation (S46), the states which may lead to successful outputs in Emma’s device include: $a_{s,t_{0}}^{\dagger}a_{u,t_{1}}^{\dagger}a_{v,t_{0}}^{\dagger}$, $a_{s,t_{0}}^{\dagger}a_{u,t_{1}}^{\dagger}a_{v,t_{1}}^{\dagger}$, $a_{s,t_{0}}^{\dagger}a_{u,t_{1}}^{\dagger}a_{w,t_{1}}^{\dagger}$, $a_{s,t_{0}}^{\dagger}a_{u,t_{1}}^{\dagger}a_{w,t_{2}}^{\dagger}$, $a_{s,t_{0}}^{\dagger}a_{u,t_{2}}^{\dagger}a_{v,t_{0}}^{\dagger}$, $a_{s,t_{1}}^{\dagger}a_{u,t_{1}}^{\dagger}a_{w,t_{1}}^{\dagger}$, $a_{s,t_{1}}^{\dagger}a_{v,t_{0}}^{\dagger}a_{w,t_{1}}^{\dagger}$, $a_{u,t_{1}}^{\dagger}a_{v,t_{1}}^{\dagger}a_{w,t_{1}}^{\dagger}$, $a_{u,t_{2}}^{\dagger}a_{v,t_{0}}^{\dagger}a_{w,t_{1}}^{\dagger}$, $a_{s,t_{0}}^{\dagger}a_{v,t_{0}}^{\dagger}a_{w,t_{1}}^{\dagger}$, $a_{s,t_{0}}^{\dagger}a_{v,t_{0}}^{\dagger}a_{w,t_{2}}^{\dagger}$, $a_{u,t_{1}}^{\dagger}a_{v,t_{0}}^{\dagger}a_{w,t_{1}}^{\dagger}$, $a_{s,t_{0}}^{\dagger}a_{u,t_{2}}^{\dagger}a_{w,t_{1}}^{\dagger}$, $a_{s,t_{0}}^{\dagger}a_{v,t_{1}}^{\dagger}a_{w,t_{1}}^{\dagger}$, $a_{s,t_{1}}^{\dagger}a_{u,t_{1}}^{\dagger}a_{v,t_{0}}^{\dagger}$ and $a_{u,t_{1}}^{\dagger}a_{v,t_{0}}^{\dagger}a_{w,t_{2}}^{\dagger}$. Their coefficients are all $16/\left(2^{3}\cdot2^{3}\cdot\sqrt{2^{3}}\right)$. In the former 12 terms, each one corresponds to 2 detection modes. In the later 4 terms, each one corresponds to 1 detection mode according to Table 1. When the input state is $a_{a,t_{0}}^{\dagger}a_{b,t_{0}}^{\dagger}a_{c,t_{0}}^{\dagger}|0\rangle$ the output probability of Emma’s device is

$\left(16/2^{3}\cdot2^{3}\cdot\sqrt{2^{3}}\right)^{2}\times\left(12\times2+4\right)\eta_{a}\eta_{b}\eta_{c}\left(1-\eta_{d}\right)Y_{0}\left(1-Y_{0}\right)^{12}$ (S47)

Similarly, we can obtain the output probabilities of other participants’ photons counts under different inputs state. So, the gain in this case is

$\begin{matrix} 1/256\left\{34\left[\eta_{a}\eta_{b}\eta_{c}\left(1-\eta_{d}\right)+\eta_{a}\eta_{b}\eta_{d}\left(1-\eta_{c}\right)\right]+\right. \\ \begin{matrix} & \end{matrix}\left.15\left[\eta_{a}\eta_{c}\eta_{d}\left(1-\eta_{b}\right)+\eta_{b}\eta_{c}\eta_{d}\left(1-\eta_{a}\right)\right]\right\}Y_{0}\left(1-Y_{0}\right)^{12} \end{matrix}$ (S48)

In this case the error will occur with probability

$\begin{matrix} 1/256\left\{17\left[\eta_{a}\eta_{b}\eta_{c}\left(1-\eta_{d}\right)+\eta_{a}\eta_{b}\eta_{d}\left(1-\eta_{c}\right)\right]+\right. \\ \begin{matrix} & \end{matrix}\left.7\left[\eta_{a}\eta_{c}\eta_{d}\left(1-\eta_{b}\right)+\eta_{b}\eta_{c}\eta_{d}\left(1-\eta_{a}\right)\right]\right\}Y_{0}\left(1-Y_{0}\right)^{12} \end{matrix}$ (S49)

(5) Case 5: outputs deriving from four photon counts

In this case, there exist successful outputs when the input state is one of $|0010\rangle\text{，}|0001\rangle\text{，}|1101\rangle$ or $|1110\rangle$. State $|0001\rangle$ can be projected into states $|\text{W}_{4,0}\rangle$ or $|\text{W}_{4,1}\rangle$ with probability $1/4$. Let the identification probabilities of $|\text{W}_{4,0}\rangle$ and $|\text{W}_{4,1}\rangle$ be $D_{p0}$ and $D_{p1}$, respectively. When input state is $|0001\rangle$ the output probability is

$1/4\eta_{a}\eta_{b}\eta_{c}\eta_{d}\left(D_{p0}+D_{p1}\right)\left(1-Y_{0}\right)^{12}$ (S50)

So, the output probability in the case is

$\begin{matrix} 1/16\times4\times1/4\eta_{a}\eta_{b}\eta_{c}\eta_{d}\left(D_{p0}+D_{p1}\right)\left(1-Y_{0}\right)^{12} \\ =1/16\eta_{a}\eta_{b}\eta_{c}\eta_{d}\left(D_{p0}+D_{p1}\right)\left(1-Y_{0}\right)^{12} \end{matrix}$ (S51)

There is no error in this case.

By adding all output probabilities in five cases together, the gain is

$\begin{matrix} \text{Q}_{1}\text{=8}\left(1-\eta_{a}\right)\left(1-\eta_{b}\right)\left(1-\eta_{c}\right)\left(1-\eta_{d}\right)Y_{0}^{4}\left(1-Y_{0}\right)^{12}+ \\ \begin{matrix} & \end{matrix}1/16\left\{52\left[\eta_{a}\left(1-\eta_{b}\right)\left(1-\eta_{c}\right)\left(1-\eta_{d}\right)+\eta_{b}\left(1-\eta_{a}\right)\left(1-\eta_{c}\right)\left(1-\eta_{d}\right)\right]\right.+ \\ \begin{matrix} & \end{matrix}38\left.\left[\eta_{c}\left(1-\eta_{a}\right)\left(1-\eta_{b}\right)\left(1-\eta_{d}\right)+\eta_{d}\left(1-\eta_{a}\right)\left(1-\eta_{b}\right)\left(1-\eta_{c}\right)\right]\right\}Y_{0}^{3}\left(1-Y_{0}\right)^{12}+ \\ \begin{matrix} & \end{matrix}1/16\left\{16\eta_{a}\eta_{b}\left(1-\eta_{c}\right)\left(1-\eta_{d}\right)\right.+10\left[\eta_{a}\eta_{c}\left(1-\eta_{b}\right)\left(1-\eta_{d}\right)+\eta_{b}\eta_{d}\left(1-\eta_{a}\right)\left(1-\eta_{c}\right)\right]+ \\ \begin{matrix} & \end{matrix}9\left[\eta_{a}\eta_{d}\left(1-\eta_{b}\right)\left(1-\eta_{c}\right)+\eta_{b}\eta_{c}\left(1-\eta_{a}\right)\left(1-\eta_{d}\right)\right]+\left.8\eta_{c}\eta_{d}\left(1-\eta_{a}\right)\left(1-\eta_{b}\right)\right\}Y_{0}^{2}\left(1-Y_{0}\right)^{12}+ \\ \begin{matrix} & \end{matrix}1/256\left\{34\left[\eta_{a}\eta_{b}\eta_{c}\left(1-\eta_{d}\right)+\eta_{a}\eta_{b}\eta_{d}\left(1-\eta_{c}\right)\right]+\right. \\ \begin{matrix} & \end{matrix}\left.15\left[\eta_{a}\eta_{c}\eta_{d}\left(1-\eta_{b}\right)+\eta_{b}\eta_{c}\eta_{d}\left(1-\eta_{a}\right)\right]\right\}Y_{0}\left(1-Y_{0}\right)^{12}+ \\ \begin{matrix} & \end{matrix}1/16\eta_{a}\eta_{b}\eta_{c}\eta_{d}\left(D_{p0}+D_{p1}\right)\left(1-Y_{0}\right)^{12} \end{matrix}$ (S52)

, and the QBER is

$\begin{matrix} e_{1}=4\left(1-\eta_{a}\right)\left(1-\eta_{b}\right)\left(1-\eta_{c}\right)\left(1-\eta_{d}\right)Y_{0}^{4}\left(1-Y_{0}\right)^{12}+ \\ \begin{matrix} & \end{matrix}1/16\left\{26\left[\eta_{a}\left(1-\eta_{b}\right)\left(1-\eta_{c}\right)\left(1-\eta_{d}\right)+\eta_{b}\left(1-\eta_{a}\right)\left(1-\eta_{c}\right)\left(1-\eta_{d}\right)\right]\right.+ \\ \begin{matrix} & \end{matrix}19\left.\left[\eta_{c}\left(1-\eta_{a}\right)\left(1-\eta_{b}\right)\left(1-\eta_{d}\right)+\eta_{d}\left(1-\eta_{a}\right)\left(1-\eta_{b}\right)\left(1-\eta_{c}\right)\right]\right\}Y_{0}^{3}\left(1-Y_{0}\right)^{12}+ \\ \begin{matrix} & \end{matrix}1/32\left\{16\eta_{a}\eta_{b}\left(1-\eta_{c}\right)\left(1-\eta_{d}\right)\right.+10\left[\eta_{a}\eta_{c}\left(1-\eta_{b}\right)\left(1-\eta_{d}\right)+\eta_{b}\eta_{d}\left(1-\eta_{a}\right)\left(1-\eta_{c}\right)\right]+ \\ \begin{matrix} & \end{matrix}9\left[\eta_{a}\eta_{d}\left(1-\eta_{b}\right)\left(1-\eta_{c}\right)+\eta_{b}\eta_{c}\left(1-\eta_{a}\right)\left(1-\eta_{d}\right)\right]+\left.8\eta_{c}\eta_{d}\left(1-\eta_{a}\right)\left(1-\eta_{b}\right)\right\}Y_{0}^{2}\left(1-Y_{0}\right)^{12}+ \\ \begin{matrix} & \end{matrix}1/256\left\{17\left[\eta_{a}\eta_{b}\eta_{c}\left(1-\eta_{d}\right)+\eta_{a}\eta_{b}\eta_{d}\left(1-\eta_{c}\right)\right]+\right. \\ \begin{matrix} & \end{matrix}\left.7\left[\eta_{a}\eta_{c}\eta_{d}\left(1-\eta_{b}\right)+\eta_{b}\eta_{c}\eta_{d}\left(1-\eta_{a}\right)\right]\right\}Y_{0}\left(1-Y_{0}\right)^{12} \end{matrix}$. (S53)

We assume that four optical fiber links and four detectors are identical, i.e. $\eta_{a}=\eta_{c}=\eta_{d}=\eta_{b}=\eta$. So, equations (S52) and (S53) can be simplified as

$\begin{matrix} Q_{1}=\left(1-Y_{0}\right)^{12}\left[1024\left(1-\eta\right)^{4}Y_{0}^{4}+1440\eta\left(1-\eta\right)^{3}Y_{0}^{3}+496\eta^{2}\left(1-\eta\right)^{2}Y_{0}^{2}+\right. \\ \begin{matrix} & \end{matrix}49\left.\eta^{3}\left(1-\eta\right)Y_{0}+8\left(D_{p0}+D_{p1}\right)\eta^{4}\right]/128 \end{matrix}$ (S54)

$\begin{matrix} e_{1}=\frac{1}{16Q_{1}}\left(1-Y_{0}\right)^{12}\left[64\left(1-\eta\right)^{4}Y_{0}^{4}+90\eta\left(1-\eta\right)^{3}Y_{0}^{3}+31\eta^{2}\left(1-\eta\right)^{2}Y_{0}^{2}+\right. \\ \begin{matrix} & \end{matrix}\left.3\eta^{3}\left(1-\eta\right)Y_{0}\right] \end{matrix}$ (S55)

1. Verstraete, F., Dehaene, J., De Moor, B. & Verschelde, H. Four qubits can be entangled in nine different ways. *Phys. Rev. A* **65,** 052112 (2002).
